# 

**Published:** 1847-07

**Authors:** 


### BRITISH AND FOREIGN MEDICAL REVIEW

OR

### QUARTERLY JOURNAL

OF

### PRACTICAL MEDICINE AND SURGERY

---

EDITED BY

JOHN FORBES M.D. F.R.S. F.G.S.

---

S.G.O.L. INDEXED

VOL. XXIV.

JULY—OCTOBER 1847

Surgeon Genl's Office LIBRARY 34454 Washington, D.C.

LONDON
JOHN CHURCHILL PRINCES STREET SOHO
MDCCCXLVII.

W1
BR34

C AND J. ADLARD, PRINTERS, BARTHOLOMEW CLOSE.

*On the* 1*st of January,* 1848, *will be published,*

**PRICE SIX SHILLINGS,**

THE FIRST NUMBER OF

THE

### BRITISH AND FOREIGN MEDICO-CHIRURGICAL REVIEW,

OR

### Quarterly Journal of Practical Medicine.

---

THE experience of the last Twelve Years having demonstrated that a remunerative sale cannot be obtained for two Quarterly Journals exclusively devoted to Medical Criticism, the respective Proprietors of the BRITISH AND FOREIGN MEDICAL REVIEW and of the MEDICO-CHIRURGICAL REVIEW, have agreed to combine their separate interests, with a view to establish a single Journal upon a comprehensive, efficient, and permanent basis.

The new Review will appear under the combined Titles of its predecessors; and will also combine, it is designed, the best features of both, in a manner that shall entitle it to yet more general acceptance with the Profession than they have singly succeeded in gaining. The high and independent tone, and the rich store of information derived from original research, which have so honorably characterized the BRITISH AND FOREIGN MEDICAL REVIEW, will be maintained without abatement or diminution; whilst the full analyses of valuable contributions to Medical Literature, pervaded by a spirit of moderate and impartial criticism, which have long rendered the MEDICO-CHIRURGICAL REVIEW so useful to a large class of readers, will continue to be presented, whenever their appearance shall be consistent with justice to the Author, as well as beneficial to the Reader.

The Journal will be composed of REVIEWS of such works as may either demand a full critical examination or may be valuable subjects for analysis, and of such as may serve as texts for original disquisitions by writers possessed of peculiar acquaintance with the topics of which they may treat;—of BIBLIOGRAPHICAL NOTICES of publications of less pretension or more remotely connected with its main objects;—and of an ample PERISCOPE, comprising analytical selections from the most important articles in Foreign Journals, and occasionally from the British Periodicals, classified under the several departments of Anatomy and Physiology (Human and Comparative), Organic Chemistry, Pathology (General and Special), Practical Medicine, Surgery, Midwifery, Pharmacy, Forensic Medicine, and Statistics.

The greatest pains will thus be taken to render the BRITISH AND FOREIGN MEDICO-CHIRURGICAL REVIEW a full and accurate record of the progress of Medical Science and Art in every part of the world. It will never be forgotten that the work is intended for the *Practitioner;* and that its success must entirely depend upon its adaptation to *his* wants. But the intimate connexion between skilful practice and scientific knowledge,—a connexion which is every day becoming more obvious,—requires that its scope should not be limited to the mere accumulation of practical details, but that it should aim to bring these into relation with scientific principles, and should take cognizance of every inquiry which directly tends to the advancement of knowledge respecting the structure and actions, natural and morbid, of the Human frame.

As a guarantee that these objects will be efficiently carried out, the Proprietors of the New Journal have the satisfaction of being able to state that they will have the advantage of the continued services of the most valued Contributors to both its predecessors; and that its General Management has been undertaken by gentlemen who have been long and honorably connected with Medical Literature.

In conclusion, they would beg to state, that whilst the British and Foreign Medico-Chirurgical Review may be regarded as a New Series of each of the Journals whose place it will occupy, it will from its commencement assume an entirely independent position. Its Conductors neither hold themselves bound by the opinions advanced in the pages of its predecessors; nor admit themselves to be in the least degree responsible for any of the critical awards there bestowed. They stand upon their own ground, and claim to be tried by their own acts alone. It will be their constant object to administer justice without fear or favour; but they will not forget that justice should be tempered with mercy, in every case but that of unprincipled and ignorant presumption; and it will be their special endeavour to avoid that carping spirit of detraction, which brings into full view the pettiest blemishes, whilst it casts the most exalted merit into the shade. Without assuming to themselves a more than human infallibility, they will honestly endeavour to give a true direction to the judgment of the profession; and it will be their highest satisfaction to find their decisions confirmed by that most authoritative tribunal.

---

The British and Foreign Medico-Chirurgical Review will be published regularly on the 1st day of *January*, *April*, *July*, and *October*. Each Number will contain eighteen sheets, or 288 pages; and two Numbers will form a Volume.

---

LONDON:

JOHN CHURCHILL, PRINCES STREET, SOHO,

AND

SAMUEL HIGHLEY, 32, FLEET STREET;

*To whom Communications for the Editors and Books for Review are to be addressed.*

Sold also by McLachlan & Stewart, and Sutherland & Knox, Edinburgh; Hodges & Smith, and Fannin & Co., Dublin.

C. AND J. ADLARD, PRINTERS, BARTHOLOMEW CLOSE.

### NEW WORKS IN MEDICINE AND SURGERY.

A PRACTICAL TREATISE on the DISEASES PECULIAR to WOMEN, Illustrated by Cases derived from Hospital and Private Practice. By SAMUEL ASHWELL, M.D., late Obstetric Physician and Lecturer to Guy's Hospital. 2d Edition, 8vo. Price 21s.

A SYSTEM of PRACTICAL SURGERY. By W. FERGUSSON, F.R.S.E. With numerous Illustrations on Wood. Second Edition. Fcap. 8vo, cloth, 12s. 6d.

A TREATISE on DISLOCATIONS and FRACTURES of the JOINTS. By Sir ASTLEY COOPER, Bart., F.R.S. Edited by BRANSBY B. COOPER, F.R.S. 8vo, cloth, 20s.

Sir Astley Cooper left very considerable additions in MS. for the express purpose of being introduced into this Edition.

A TREATISE on RUPTURES. By W. LAWRENCE, F.R.S. The Fifth Edition, considerably enlarged. 8vo, cloth, 16s.

ON DISEASES of the LIVER. By Dr. BUDD, F.R.S. Illustrated with Coloured Plates and Engravings on Wood. 8vo, cloth, 14s.

PARIS'S PHARMACOLOGIA. 9th Edition. Being an extended inquiry into the operations of Medicinal Bodies, upon which are founded the THEORY and ART of PRESCRIBING. Rewritten, in order to incorporate the latest Discoveries in Physiology, Chemistry, and Materia Medica. By J. A. PARIS, M.D., F.R.S., President of the Royal College of Physicians. 8vo, 20s.

PRACTICAL OBSERVATIONS and SUGGESTIONS in MEDICINE. By MARSHALL HALL, M.D., F.R.S. Post 8vo, cloth, 8s. 6d.

DITTO. *Second Series.* 8s. 6d.

PRINCIPLES of HUMAN PHYSIOLOGY. By W. B. CARPENTER, M.D., F.R.S. With numerous Illustrations on Steel and Wood. Third Edition. One thick 8vo vol., 21s.

RESEARCHES and OBSERVATIONS on SCROFULOUS DISEASES. By J. G. LUGOL. Translated, with an Appendix, by W. H. RANKING, M.D. 8vo, cloth, 10s. 6d.

SPRATT'S OBSTETRIC TABLES. 4th Edition. These Tables are designed on a similar principle to Mr. Tuson's Anatomical Plates,—the Views being disposed in hinged layers, the raising of which shows a progressive advance in the subject under consideration. There are 19 Tables, most of which are made to furnish several successive views by means of this mechanical adaptation. 1 vol. 4to, price 28s. coloured.

THE DIAGNOSIS, PATHOLOGICAL INDICATIONS and TREATMENT of URINARY DEPOSITS. By GOLDING BIRD, M.D., F.R.S. With Engravings on Wood. Second Edition. Post 8vo, cloth, 8s. 6d.

THE PHYSIOLOGY of INFLAMMATION and the HEALING PROCESS. By BENJAMIN TRAVERS, F.R.S., Surgeon Extraordinary to the Queen, &c. 8vo, 7s.

TRANSACTIONS of the MEDICAL SOCIETY of LONDON; containing Papers on the Use of the Microscope in Anatomy, Physiology, and Pathology; by Mr. THOS. BELL, F.R.S., &c.—The Cause and Treatment of Stammering; by Mr. J. BISHOP, F.R.S.—On the Nervous System, particularly the Excito-motory, or Reflex-function; by Mr. G. PILCHER.—On the Prevention and Treatment of Apoplexy and Hemiplegia; by Dr. MARSHALL HALL.—On the Incubation of Insanity; by Dr. FORBES WINSLOW.—Also Papers by Dr. J. R. Bennett; Mr. Bryant; Mr. Crisp; Mr. Dendy; Dr. Garrod; Mr. Headland; Mr. Hutchinson; Mr. Linnecar; Mr. Robarts; Mr. Stedman; Dr. T. Thomson; Dr. Waller, &c. 8vo, Plates, 9s.

### THE BRITISH AND FOREIGN MEDICAL REVIEW.

**NO. XLVII.—JULY, 1847.**

### PART FIRST.—Analytical and Critical Reviews.

| | | PAGE |
|---|---|---|
| 1. | SEGUIN and SCOTT on the Moral Treatment, Hygiene, and Education of Idiots | 1 |
| 2. | Dr. KRAMER on the Statistics of Diseases of the Ear | 22 |
| 3. | HENOCH and ROMBERG's Clinical Observations | 41 |
| 4. | DR. PRICHARD on the Physical and Natural History of Mankind | 49 |
| 5. | HEUSINGER's Comparative Pathology | 81 |
| 6. | Dr. CASPER on Medical Statistics: Influence of the Seasons | 91 |
| 7. | Dr. GUNSBURG's Pathological Histology | 118 |
| 8. | Dr. BAUMGAERTNER on Physiology and Practical Medicine | 128 |
| 9. | Dr. SEYMOUR's Thoughts on several Severe Diseases of the Human Body | 139 |
| 10. | Dr. CONOLLY on the Construction and Government of Lunatic Asylums | 156 |
| 11. | MEHLISS on the Diseases of the Diaphragm | 166 |
| 12. | UNZER's Principles of Animal Physiology | 181 |
| 13. | Drs. SANKEY and M'ARTHUR's Medical Instructions for Merchant Seamen | 212 |
| 14. | Mr. GREEN's Mental Dynamics—Hunterian Oration | 216 |
| 15. | MR. HENFREY's Outlines of Botany | 223 |
| 16. | Dr. MAYO on the Relations of Insanity, Crime, and Punishment | 225 |
| 17. | PRUS, WHITE, &c., on Plague and Quarantine | 242 |
| 18. | LIEBIG's New Researches on the Chemistry of Food | 264 |
| 19. | Dr. ALDERSON on Diseases of the Stomach and Alimentary Canal | 273 |
| 20. | VOGEL on the Laws regarding the Mixing of Fluids in the Body, &c. | 276 |

### PART SECOND.—Bibliographical Notices.

| | | |
|---|---|---|
| 1. | Dr. DOWLER on the Post-mortem Contractility of the Muscles | 279 |
| 2. | Mr. HUNT on Diseases of the Skin | 280 |
| 3. | Professor ANSTED's Ancient World | 281 |
| 4. | Dr. WRIGHT's Introductory Lecture | 282 |
| 5. | Mr. GLAISHER's Hygrometrical Tables | 283 |
| 6. | Dr. BOOTH on Education and Educational Institutes | 284 |
| 7. | Mr. KENNEDY on Famine and Fever in Ireland | 285 |
| 8. | Mr. CHARLTON's Account of Scarlatina in Newcastle | 286 |
| 9. | Dr. VON BIBRA on Zoochemical Substances | ib. |
| 10. | Mr. REDFORD on Body and Soul | 287 |
| 11. | BELLINI on Metastasis | 288 |

No. XLVIII WILL BE PUBLISHED ON THE 1st OF OCTOBER, 1847.

COMMUNICATIONS, JOURNALS, *and* BOOKS FOR REVIEW, *to be forwarded* (*Carriage paid*) *to the care of* MR. JOHN CHURCHILL, PRINCES STREET, SOHO; *or direct to the* EDITOR, 12, OLD BURLINGTON STREET, LONDON.

